# Molecular Basis of Bcl-X_L_-p53 Interaction: Insights from Molecular Dynamics Simulations

**DOI:** 10.1371/journal.pone.0026014

**Published:** 2011-10-19

**Authors:** Nagakumar Bharatham, Seung-Wook Chi, Ho Sup Yoon

**Affiliations:** 1 Division of Structural Biology and Biochemistry, School of Biological Sciences, Nanyang Technological University, Singapore; 2 Medical Proteomics Research Center, KRIBB, Daejeon, Republic of Korea; University of South Florida College of Medicine, United States of America

## Abstract

Bcl-X_L_, an antiapoptotic Bcl-2 family protein, plays a central role in the regulation of the apoptotic pathway. Heterodimerization of the antiapoptotic Bcl-2 family proteins with the proapoptotic family members such as Bad, Bak, Bim and Bid is a crucial step in the apoptotic regulation. In addition to these conventional binding partners, recent evidences reveal that the Bcl-2 family proteins also interact with noncanonical binding partners such as p53. Our previous NMR studies showed that Bcl-X_L_: BH3 peptide and Bcl-X_L_: SN15 peptide (a peptide derived from residues S15-N29 of p53) complex structures share similar modes of bindings. To further elucidate the molecular basis of the interactions, here we have employed molecular dynamics simulations coupled with MM/PBSA approach. Bcl-X_L_ and other Bcl-2 family proteins have 4 hydrophobic pockets (p1–p4), which are occupied by four systematically spaced hydrophobic residues (h1–h4) of the proapoptotic Bad and Bak BH3 peptides. We observed that three conserved hydrophobic residues (F19, W23 and L26) of p53 (SN15) peptide anchor into three hydrophobic pockets (p2–p4) of Bcl-X_L_ in a similar manner as BH3 peptide. Our results provide insights into the novel molecular recognition by Bcl-X_L_ with p53.

## Introduction

Apoptosis or programmed cell death is a key regulatory process involved in major biological pathways in which its dysregulation is linked to cancer, autoimmunity, and neurodegenerative disorders [1]. The Bcl-2 family proteins regulate and mediate the mitochondrial outer membrane permeabilization, a crucial event in the mitochondrial pathway of apoptosis in vertebrates [2]–[5]. The regulation of apoptosis is governed largely by interactions between the pro-survival and pro-death members of the Bcl-2 protein family [6]. Some members of this family (e.g., Bax, Bak, and Bid) promote apoptosis, while others such as Bcl-X_L_, Bcl-2 and Bcl-w work against programmed cell death [7], [8]. The Bcl-2 family proteins are characterized by regions of specific sequence homology named as Bcl-2 homology (BH) motifs that number from 1 to 4 and are critical for function [9]. Especially α-helical BH3 motif of proapoptotic proteins occupy and form strong interactions with hydrophobic groove of antiapoptotic Bcl-2 family proteins which leads to the activation of the essential death mediators Bax and Bak, thereby committing cells to apoptosis [10]–[15].

p53, a key tumor suppressor protein also termed as “the guardian of the genome”, plays a key role in cellular stress response pathway [16], [17]. It is found to be mutated or lost in more than 50% of all human cancers indicating its crucial functions in controlling tumor formation [18]. Under normal conditions, p53 is quiescent and present at basal levels. Upon cellular stress, DNA damage and hypoxia, it is upregulated and induces pathways that cause cell cycle arrest, DNA repair, cellular senescence, differentiation and apoptosis [19], [20]. The proapoptotic activity of the tumor suppressor protein p53 is controlled by a number of protein–protein interactions that constitute a network of negative and positive regulators [21]. The central part of this network is the interaction with the oncogenic protein MDM2 via the N-terminal transactivation domain (TAD) and the central DNA-binding domain (DBD) [22], [23]. Binding of the E3 ubiquitin ligase, MDM2 to the tumor suppressor protein, targets p53 for proteosomal degradation [24].

While the transcription-dependent mechanism of p53 has been extensively studied [25], evidence supporting the transcription-independent apoptotic activity of p53 has emerged in recent years [26], [27]. This suggests that p53 could be localized in the outer membrane of mitochondria and execute the transcription-independent apoptotic cell death in response to death signals [28], [29]. Recent studies on the transcription-independent mitochondrial p53 apoptotic pathway provided valuable information [30]–[34]. It was demonstrated that PUMA, a proapoptotic BH3 only protein releases p53 from Bcl-X_L_/p53 complex and allows Bax or Bak to induce mitochondrial permeability [33] while p53 upregulated Bad, another proapoptotic protein by forming a complex in the mitochondria thereby inducing apoptosis [34]. These studies revealed that Bcl-2 family proteins are the binding targets for p53 and results in a transcription-independent apoptotic activity.

The structures of Bcl-X_L_ in complex with Bad or Bak proapoptotic BH3 peptides offer detailed structural information on the binding interface between the protein and complementary peptide residues [35], [36], revealing possible hydrophobic pockets and important interactions which provide a molecular basis to develop sub-nanomolar range inhibitors [37]. Similarly, studies on MDM2/p53 complex studies also contributed to the designing of antagonists to disturb p53-MDM2 interactions [38]–[40]. Based on this structural information, several p53 peptidomimetics were developed [41]–[43]. Computational studies provided insightful information into the molecular recognition between p53 and MDM2 [44]–[48]. In previous NMR studies we have attempted to understand the binding properties of p53 with its non-conventional partner Bcl-X_L_, and based on those observations we developed a complex model [49], [50]. In the present study we have performed several molecular dynamics simulations on Bcl-X_L_/BH3 peptides (Bad, Bak), MDM2/p53 and Bcl-X_L_/p53 (SN15) peptide complexes and examined the common interaction pattern of these peptides with their binding partners using multiple conformations. Binding free energy calculations and residual decomposition analyses were opted to understand recognition process of these peptides by Bcl-X_L_.

## Methods

### Starting structures

The initial coordinates of MDM2/p53 and Bcl-X_L_/BH3 peptide complexes were obtained from the Protein Data Bank. The wild type human p53 (residues 17–29) and MDM2 (residues 25–109) complex crystal structure with PDB ID: 1YCR was utilized [38]. Two proapoptotic proteins Bad and Bak BH3 peptides in complex with human Bcl-X_L_ (PDB ID 1G5J and 1BXL respectively) were considered as starting structures for Bcl-X_L_/BH3 peptide complex simulations [35], [36]. The Bcl-X_L_/SN15 peptide complex coordinates were taken from our previous model developed by using HADDOCK based on chemical shift perturbations observed for Bcl-X_L_ upon complex formation with SN15 peptide [50].The Bcl-X_L_/Bak BH3 peptide complex structure (PDB ID: 1BXL) was utilized to develop the Bcl-X_L_/SN15 peptide complex model. The N- and C-terminus residues of the proteins were capped with ACE and NME, accordingly to keep them neutral at the time of simulation. The N-terminal end alone was capped for peptide in the MDM2/p53 complex, based on previous computational studies [45]–[48].

### Molecular dynamics simulations

All the MD simulations were performed using GROMACS software (version 4.0.5) [51], [52] with the Amber99 force field. The MD simulation protocol that we used is as follows. Hydrogens were added and the protonation state of ionizable groups was chosen appropriate to pH 7.0. Each system was inserted in a water box of TIP3P water, which extended at least 12 Å away from any given protein atom. All systems were neutralized by adding counter ions and replacing the overlapping solvent molecules. The size of each system utilized for the simulations are represented in Table 1. All simulations were run under periodic boundary conditions with NPT ensemble by using Berendsen's coupling algorithm for maintaining the temperature (300 K) and the pressure constant (1 bar). The SHAKE algorithm with a tolerance of 10^−5^ Å was applied to fix all bonds containing hydrogen atoms. The electrostatic interactions were calculated by using the Particle-mesh Ewald (PME) algorithm, with interpolation order of 4 and a grid spacing of 0.1 nm and the van der Waals forces were treated by using a cutoff of 10 Å. A 2-fs time step was used to integrate the equations of motion. The systems were subjected to steepest descent energy minimization for 2,000 steps. Then the protein backbone was frozen and the solvent molecules with counter ions were allowed to move during a 200 ps position restrained MD run. The final production run continued for 10 or 15 ns (Table 1) and the coordinates were stored every 1 ps. The purpose of the simulations was to generate multiple structures around the initial experimental/model structure to improve the statistical sampling for binding free energy (ΔG_bind_) calculations using MM/PBSA method.

**Table 1:** Summary of MD simulations performed in present study.

System	Initial coordinates	No. of atoms	Simulation length
MDM2/p53	1YCR	18,671	10 ns
Bcl-X_L_/Bad	1G5J	35,627	15 ns
Bcl-X_L_/Bak	1BXL	35,520	15 ns
Bcl-X_L_/SN15	Model	34,103	15 ns
Bcl-X_L_/SN15W23A	Model	34,100	10 ns

### Binding free Calculations

The binding energy of protein-peptide complexes was calculated by using MM/GBSA (for MDM2/p53) and MM/PBSA (for remaining simulations) incorporated in Amber9 package [53]–[55]. MDM2/p53 simulation was utilized to benchmark our MD simulation protocol as this system is well studied by computational simulations. The trajectory files generated by production run of different systems are converted into individual PDB files by trajconv method incorporated in GROMACS analysis suite. For MDM2/p53 and Bcl-X_L_/SN15W23A mutant simulation (10 ns each) 2500 snapshots were considered at the time intervals of 2 ps from the last 5 ns production runs. For remaining three 15 ns simulations (Bcl-X_L_ with Bad, Bak and SN15 peptides) 5000 snapshots were chosen from the last 10 ns. These selected snapshots were utilized to calculate the enthalpy contributions of protein-peptide complexes and every 100^th^ snapshot of selected frames (25 or 50 frames for 2500 or 5000 snapshots, respectively) was used to calculate entropy terms using normal mode analysis (Nmode module of Amber) and averaged [56]. The dielectric constants for solute and solvent set as 1.0 and 80.0, respectively. The solvent accessible surface area (SASA) was computed with molsurf module in Amber9, using a probe radius of 1.4 Å [57]. The surface tension proportionality constant and the free energy of nonpolar solvation of a point solute were set to 0.00542 kcal/Å^2^ and 0.92 kcal/mol, respectively. A single trajectory method was used in MM-GBSA/PBSA binding free energy calculations. Residual decomposition analysis which is incorporated in the Amber9 software package was utilized to understand the important interactions between protein-peptide complexes and also to select the crucial hot spot interactions which are essential for the complex formation and stability. Every 50^th^ snapshot of selected frames (50 or 100 frames for 2500 or 5000 snapshots, respectively) was used to calculate the energy decomposition for all the protein and peptide residues. All the preliminary analyses to ensure the quality of the simulations such as root mean square deviation (RMSD), root means square fluctuation (RMSF), secondary structural analyses by DSSP are carried out by GROMACS analysis programs.

## Results

### Sequence comparison of BH3 and SN15 peptides

**Figure 1:**
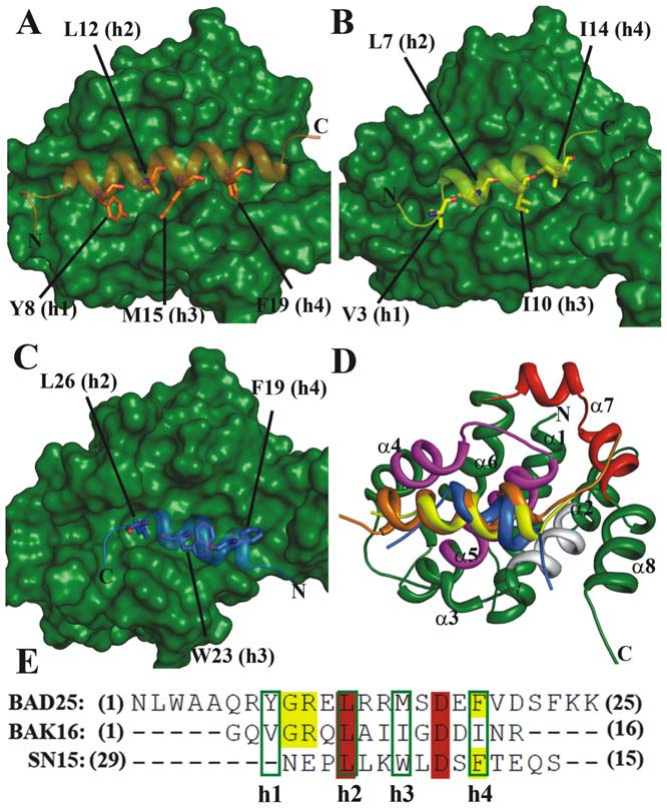
Binding orientation and sequence comparison of SN15 with BH3 peptides. The interaction pattern of Bcl-X_L_ and Bad (A), Bak (B), SN15 (C) was shown. All the three peptides represented as cartoon and Bcl-X_L_ as surface. Hydrophobic residues of the peptides which occupy the four hydrophobic pockets (p1–p4) are highlighted by sticks and labeled accordingly. All the three peptides (Bad, Bak and SN15) superimposed and the important interacting regions of Bcl-X_L_ such as BH1, BH2 and BH3 are highlighted with magenta, red and gray, respectively (D). Shown is sequence comparison between SN15 and Bad and Bak. Four important binding points (h1–h4) are highlighted by green boxes, identical residues and conserved residues are highlighted by red and yellow colors, respectively (E).

Antiapoptotic proteins such as Bcl-X_L_, Bcl-2, Bcl-w and Mcl-1 possess four conserved hydrophobic pockets (p1–p4) which provide space for four hydrophobic residues (h1–h4) present on the BH3 peptides of Bad and Bak. Sequence alignment was carried out by clustalW method [58] to obtain initial insights of similarities between the BH3 (Bad25 and Bak16) and SN15 peptides. We have failed to achieve a meaningful sequence alignment by normal procedure of taking SN15 sequence in conventional way (15SQETFSDLWKLLPEN29). Our previous NMR chemical shift perturbation experiments and HADDOCK model revealed that the binding orientation of SN15 peptide to Bcl-X_L_ is in opposite direction (29NEPLLKWLDSFTEQS15) when compared to BH3 peptides such as Bad and Bak binding orientations (Figure 1A–1D) [49], [50]. Based on these previous observations, the binding orientations of the peptides with Bcl-X_L_ were considered for sequence alignment i.e. Bad (N1→K25), Bak (G1→R16) and SN15 (N29→S15). By this attempt, the sequences aligned well and all the important hydrophobic residues that are crucial for Bcl-X_L_ binding are aligned properly (Figure 1E). These results also revealed that SN15 has three hydrophobic residues (L26, W23 and F19) which occupy three hydrophobic pockets (p2, p3 and p4).

### Preliminary analysis of MD simulations

**Figure 2:**
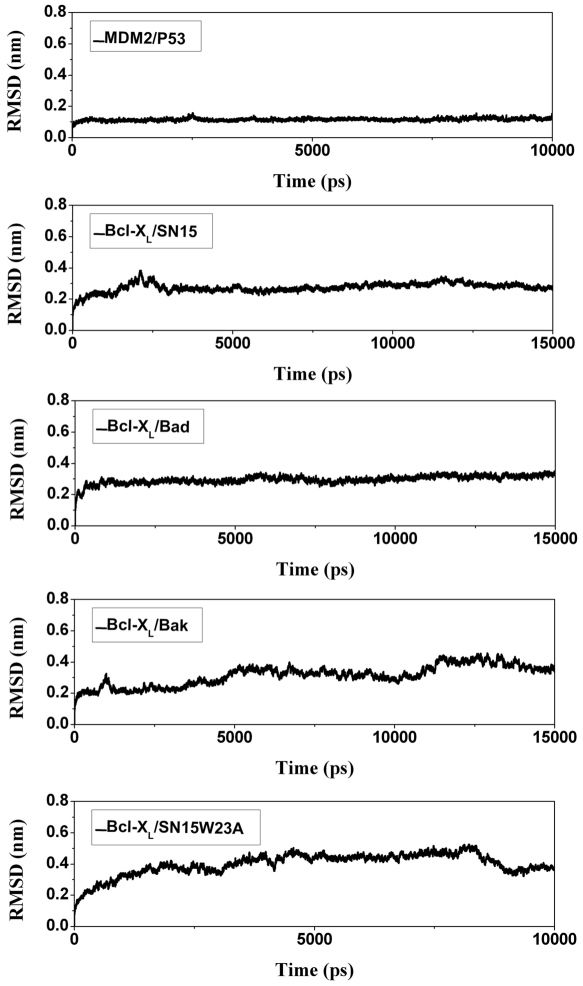
RMSD plots for five MD simulations. The Root mean square deviation (RMSD) of backbone atoms were shown with respect to initial minimized structure for all the five simulations such as MDM2/p53 (A), Bcl-X_L_/SN15 (B), Bcl-X_L_/Bad (C), Bcl-X_L_/Bak (D), and Bcl-X_L_/SN15W23A (E).

**Figure 3:**
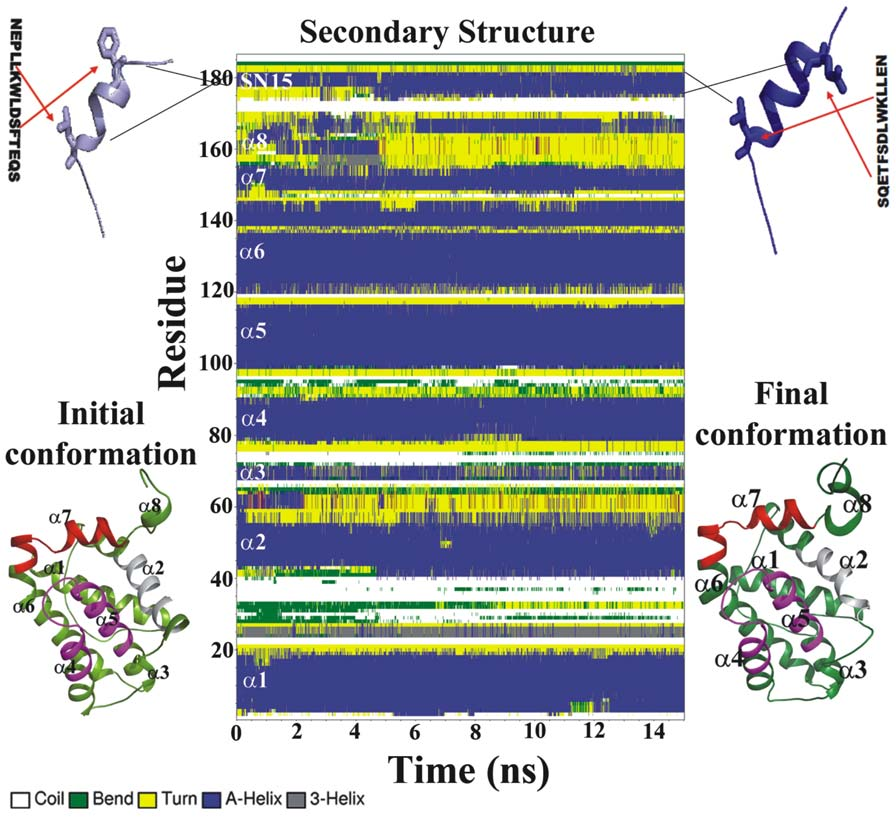
Stability of secondary structural features of Bcl-X_L_/SN15 complex. Secondary structural characteristics were calculated using DSSP for total simulation to understand the stability and changes for the Bcl-X_L_/SN15 complex. The initial (7 residues) and final (9 residues) helix length of the SN15 are represented with starting and ending residues of helix and highlighted by arrows. Initial and final frames of the protein represented as cartoon model and labeled.

Total five simulations were carried out for MDM2/p53 complex, Bcl-XL/Bad, Bcl-XL/Bak, Bcl-XL/SN15 and Bcl-XL/SN15W23A, a SN15 point mutant peptide. The MD simulations were judged to be stable as evidenced by the time dependent evaluation of backbone root mean square deviation (RMSD). The RMSD was calculated during production phase using the respective initial minimized structure as the reference structure. The average RMSD value for MDM2/P53 complex simulation was 0.12 nm which is consistent with previous computational simulation studies [45]. For the Bcl-X_L_/peptide (SN15, Bad and Bak) simulations the average RMSD values was between 0.28 nm to 0.32 nm. The Bcl-X_L_/SN15 mutant (W23A) peptide simulation showed bit higher RMSD average values (0.4 nm), which can be expected due to changes in the peptide secondary structure and overall fluctuations of protein structure. Comparatively high fluctuations were observed at the long flexible loop of Bcl-X_L_ located between α1 and α2 and also other loops which connect the α-helices. These observations suggest that there is no significant structural drift in each system during the MD simulations (Figure 2). Secondary structural analysis was carried out to measure the stability of the simulations. These analysis shows that the α-helices present in the MDM2 as well as Bcl-X_L_ persist throughout the simulation time (Figure 3 and Figure S1A–S1D). The short β-strands present in the MDM2 structures also maintained their size (Figure S1A). The structural differences were observed at the loop positions that connect the α-helices due to high flexible nature. Previous experimental and computational studies demonstrated that helical content of the peptides (p53 or BH3 peptides) is directly proportional to its binding ability with partners [47], [59]. DSSP analysis revealed that all the peptides indeed preserve their helical content and in several simulations there is an increase by 2–3 residues (Figure 3 and Figure S1A–S1D). The stability and increment in the helical content is also evident from the intra-molecular hydrogen bonds between main chain atoms of the peptides (Figure S2A–S2D).

### Free energy decomposition of protein-peptide complexes and comparison

We have adopted binding free energy calculations and decomposition of residue wise contribution to understand the similarities between BH3 domains of proapoptotic proteins (Bad and Bak) and N-terminal transactivation domain of p53 (SN15). These results also facilitate us to identify the key residues of p53 which are crucial for binding with MDM2 and novel binding partner Bcl-X_L_. For the purpose of this work and to identify hot spot amino acids in protein and peptide complexes, we defined key residue as the one that makes a −1 kcal/mol contribution to the binding free energy.

### Energy calculations for MDM2 with p53 peptide

**Figure 4:**
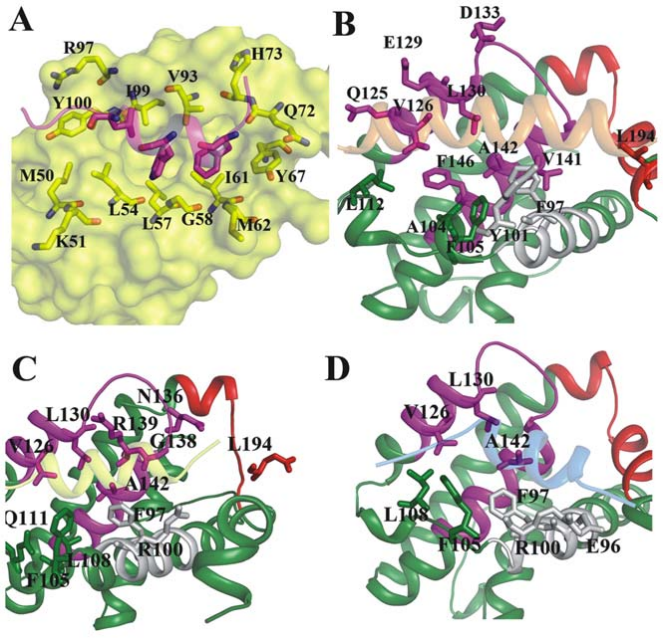
Major contributing residues of MDM2 and Bcl-X_L_ for complex formation with SN15 and BH3 peptides. Pictorial representation of important residues of MDM2 for complex formation with p53 (A). The p53 peptide is shown in magenta cartoon and MDM2 protein is highlighted by surface representation. The major contributors from both protein and peptide are represented by sticks and labeled. The Bcl-X_L_ protein and peptides Bad (B), Bak (C), SN15 (D) are represented as cartoon model and major contributing residues are shown as sticks. Important interacting regions of Bcl-X_L_ such as BH1, BH2 and BH3 are highlighted as magenta, red and gray, respectively.

Binding free energy calculations were carried out for MDM2/p53 complex to better understand the interaction pattern of p53 with MDM2 as well as to unveil the hot spot residues of p53 which are common in complex formation with MDM2 and Bcl-X_L_. This simulation results also allowed us to compare with previous computational studies and benchmark our MD simulation protocol. For this purpose we have opted MM/GBSA method to calculate the binding free energy of MDM2/p53 complex. The ΔG_bind_ of this complex was computed as −11.3 kcal/mol (Table 2 and Table 3) which is close to experimental binding free energy (−6.4 to −9.0 kcal/mol) [60], [61] and other computational studies estimates −6.9 to −16 kcal/mol [44]–[48]. The residue level contribution was carried out to extract the key residues information from both protein (MDM2) and peptide. The results suggest that the residues, F19, W23 and L26 of p53 contribute more in binding and moreover F19 is better contributor for binding than W23 (Table 4), which is consistent with previous computational observations [48]. Several hydrophobic and one or two hydrophilic MDM2 residues which are important in complex formation were also identified (Figure 4A).

**Table 2:** Bind free energy (kcal/mol) and components for different protein-peptide complexes.

System	ΔH	TΔS	ΔG_bind_	ΔG_bind_ (expt)^a^
MDM2/p53	−50.4	−39.1	−11.3	−6.4 to −9.0
Bcl-X_L_/Bad	−71.0	−53.3	−17.7	−12.7
Bcl-X_L_/Bak	−62.1	−49.8	−12.3	−8.9 to −9.3
Bcl-X_L_/SN15	−49.3	−42.0	−7.3	−4.9
Bcl-X_L_/SN15W23A	−34.8	−37.0	2.2	NB^b^

a Experimental values obtained from the previous studies [35], [36], [50], [59]–[61].

b NB; no binding [50].

**Table 3:** Components of binding free energy (in kcal/mol) of MDM2 with p53 peptide.

	MDM2/p53		MDM2		p53		Delta
	Average	Std	Average	Std	Average	Std	
ELE	−2839.2	48.6	−2282.1	43.5	−181.5	20.0	−375.6
VDW	−357.4	18.1	−274.5	16.0	−11.0	5.7	−71.9
GAS	−1322.8	51.9	−934.8	49.0	59.5	21.4	−447.5
GBSUR	35.3	0.7	33.6	0.5	10.7	0.2	−9.0
GB	−1393.3	45.8	−1329.9	39.5	−469.4	17.4	406.0
GBSOL	−1358.0	45.4	−1296.3	39.2	−458.7	17.3	397.0
GBELE	−4232.5	18.4	−3612.0	18.1	−651.0	6.4	30.5
**GBTOT**	−2680.8	35.3	−2231.1	33.7	−399.3	12.5	**−50.4**
TSTRA	16.2	0	16.0	0	14.4	0	−14.3
TSROT	16.0	0	15.9	0	13.3	0	−13.1
TSVIB	1179.9	3.0	1023.8	3.5	167.9	1.4	−11.8
**TSTOT**	1212.1	3.0	1055.7	3.5	195.5	1.4	**−39.1**
Δ**G_bind_**							**−11.3**

Electrostatic energy (ELE); van der Waals contribution (VDW); total gas phase energy (GAS); nonpolar contribution to the solvation free energy (GBSUR); the electrostatic contribution to the solvation free energy (GB); sum of nonpolar and polar contributions to solvation (GBSOL); sum of the electrostatic solvation free energy and MM electrostatic energy (GBELE); final estimated binding free energy (GBTOT); translational energy (TSTRA); rotational energy (TSROT); vibrational energy (TSVIB), total entropic contribution (TSTOT); binding free energy (**ΔG_bind_**).

**Table 4:** Residual decomposition analyses to recognize the important contributors of MDM2 and p53 peptide.

Residue (MDM2)	Energy contribution (kcal/mol)	Residue (p53)	Energy contribution (kcal/mol)
M50	−1.17	E17	−0.15
K51	−1.14	T18	−1.59
L54	−3.27	**F19** a	**−6.58**
L57	−1.13	S20	0.32
G58	−1	D21	−0.1
I61	−2.03	L22	−2.26
M62	−1.15	**W23** a	**−4.9**
Y67	−1.17	K24	0.91
Q72	−1.64	L25	−1.06
H73	−1.16	**L26** a	**−3.99**
V93	−1.65	P27	−1.95
R97	−6.61	E28	0.27
I99	−1.16	N29	−0.84
Y100	−1.81		

a Three major contributing hydrophobic residues of p53 peptide (F19, W23 and L26) are highlighted as bold.

### Binding free energy calculations and their decomposition for Bcl-X_L_/peptide complexes

The Bcl-X_L_/BH3 peptide complexes were well studied as the three dimensional structures were solved by either NMR or X-ray crystallographic methods and also by computational studies [35], [36]. But the interaction pattern between Bcl-X_L_ and p53 (SN15) and the key residues which are involved in the complex formation is not fully known. The calculated ΔG_bind_ was −17.7 and −12.3 kcal/mol for Bcl-X_L_/Bad and Bcl-X_L_/Bak complexes respectively (Table 2, Table S1 and S2). The experimental methods estimated −12.7 and −8.94 to −9.32 kcal/mol for Bcl-X_L_/Bad and Bcl-X_L_/Bak complexes respectively [35], [36]. Our previous experimental binding studies between Bcl-X_L_/SN15 peptide estimated ΔG_bind_ as −4.95 kcal/mol [50] and present computational binding free energy method estimated −7.3 kcal/mol (Table 5). These results demonstrate that computational free energies are estimated close to the experimental binding values with acceptable differences. The residual decomposition results identified that several residues of Bcl-X_L_ which are important for heterodimer formation with BH3 peptides like Bad/Bak are indeed important for complex formation with SN15 with fewer differences. The three hydrophobic residues (F19, W23 and L26) of p53 which are important in complex formation with MDM2 are also crucial for complex formation with Bcl-X_L_(Table 6).

**Table 5:** Components of binding free energy (in kcal/mol) of Bcl-X_L_ with SN15 peptide.

	Bcl-X_L_/SN15		Bcl-X_L_		SN15		Delta
	Average	Std	Average	Std	Average	Std	
ELE	−4796.6	77.2	−4659.3	78.7	−326.4	31.7	189.1
VDW	−689.7	26.1	−600.2	25.2	−12.0	5.8	−77.5
GAS	−1984.6	87.2	−2048.1	87.6	−48.1	32.5	111.6
PBSUR	58.4	1.48	57.1	1.38	11.7	0.2	−10.5
PB	−4081.4	75.0	−3465.2	79.0	−465.7	30.2	−150.5
PBSOL	−4023.0	74.3	−3408.1	78.2	−454.0	30.1	−160.9
PBELE	−8877.9	29.9	−8124.5	28.6	−792.1	6.5	38.7
**PBTOT**	−6007.6	46.4	−5456.2	44.1	−502.0	12.5	**−49.3**
TSTRA	16.7	0	16.6	0	14.5	0	−14.4
TSROT	16.9	0	16.8	0	13.4	0	−13.3
TSVIB	2110.7	8.5	1938.7	7.1	186.3	1.7	−14.3
**TSTOT**	2144.4	8.5	1972.1	7.1	214.3	1.7	**−42.0**
Δ**G_bind_**							**−7.3**

Electrostatic energy (ELE); van der Waals contribution (VDW); total gas phase energy (GAS); nonpolar contribution to the solvation free energy (PBSUR); the electrostatic contribution to the solvation free energy (PB); sum of nonpolar and polar contributions to solvation (PBSOL); sum of the electrostatic solvation free energy and MM electrostatic energy (PBELE); final estimated binding free energy (PBTOT); translational energy (TSTRA); rotational energy (TSROT); vibrational energy (TSVIB), total entropic contribution (TSTOT); binding free energy (**ΔG_bind_**).

**Table 6:** Bcl-X_L_ residues and their energy contribution for each peptide.

Residue (Bcl-XL)	Energy contribution (kcal/mol)		
	Bad	Bak	SN15
E96	-	-	−2.78
F97	−3.35	−2.5	−2.53
R100	-	−3.44	−4.76
Y101	−1.7	-	-
A104	−1.45	-	-
F105	−1.04	−2.35	−1.47
L108	-	−1.24	−2.26
Q111	-	−2.56	-
L112	−1.72	-	-
Q125	−1.03	-	-
V126	−3.46	−1.75	−1.25
E129	−2.78	-	-
L130	−3.44	−2.27	−2.11
D133	−4.65	-	-
N136	-	−2.3	-
G138	-	−1.31	-
R139	-	−5.11	-
V141	−1.08	-	-
A142	−1.28	−1.09	−1.14
F146	−1.4	−1.35	-
L194	−1.19	−1.37	-

## Discussion

### Key anchoring residues of p53 for MDM2 binding

The three hydrophobic residues (F19, W23 and L26) that occupy the hydrophobic surface of the MDM2 are crucial for the binding [62]. Previous experimental studies revealed that these residues are susceptible for mutations which show severe decrease in binding or even inactive in some cases [43]. Residue F19 of p53 which is predicted as major contributor (−6.58 kcal/mol) for MDM2 binding forms strong van der Waals contacts with I61 as well as with G58, Y67 and V93. The tryptophan residue of p53 protrudes into hydrophobic pocket formed by several conserved aliphatic hydrophobic residues like L54 and L57. The residual level energetics revealed that L54 contributes strongly from MDM2 part, this is due to both van der Waals contact with tryptophan and also stable hydrogen bond. This hydrogen bond is observed between NEε of tryptophan and main chain carbonyl of L54. Another conserved hydrophobic residue L26 of p53 interacts with I99 side chain. Our results are also consistent with previous experimental and computational findings and corroborate that these three hydrophobic residues of p53 are determinants for MDM2 binding. Apart from these three crucial interactions several other residues also helps for the firm binding such as L22, P27 and T18. The residue L22 of p53 interacts with V93 and H73 of binding partner. Proline residue (P27) present on the p53 peptide forms van der Waals interactions with Y100 of MDM2.

### Major contributors of BH3 peptides for hetero-dimerization with Bcl-X_L_

Four conserved hydrophobic pockets (p1–p4) are available in all the Bcl-2 family members like Bcl-X_L_, Bcl-2, Mcl-1 and Bcl-w proteins. Most of the residues which form these hydrophobic pockets are also conserved across the family members. All four pockets are formed by residues present on BH1 (α4 and α5 helices) majorly, BH2 (α7) and BH3 (α2) helices of Bcl-X_L_. These hydrophobic pockets provide space for the well-spaced (i, i+4, i+7 and i+11) hydrophobic residues (h1–h4) on BH3 peptides such as Bad/Bak (Figure 1A and 1B). The hydrophobic residues of BH3 peptides lock with binding partner by forming strong van der Waals interactions. Several experimental and computational studies demonstrated that these four hydrophobic residues are crucial for heterodimer formation [35], [36], [44], [63]. Residual decomposition results illustrate that these four hydrophobic residues of the Bad/Bak peptide contribute sufficiently well for binding with the Bcl-X_L_ (Table 6).

Residue Y8 of Bad peptide and V3 of Bak peptide occupies p1 hydrophobic pocket of Bcl-X_L_. This pocket is formed by F105, L112, V126 and F146 which constitutes BH1 and BH3 helices and a short α3 helix. A stable hydrogen bond interaction observed between side chain hydroxyl of Bad Y8 and main chain carbonyl of Bcl-X_L_ A104. The p2 pocket of Bcl-X_L_ is formed by F97, F105, V126, L130 as well as F146 and locks with conserved leucine residue of BH3 peptides (L12, L7 of Bad and Bak, respectively). The residue M15 of Bad peptide and I10 of Bak form van der Waals interactions with p3 hydrophobic pocket formed by F97 and A142 residues. Finally the p4 pocket formed by F97 and V141 residues is occupied by F19 (Bad) and I14 (Bak). Besides these crucial hydrophobic interactions, several other hydrophobic interactions are observed to be important for binding. A5 residue of Bad peptide contributed ∼−2.8 kcal/mol for complex formation. This residue forms close contacts with L112, S122, Q125 and V126 and make favorable contribution for binding energy. Another hydrophobic residue F23 of Bad peptide forms firm van der Waals contacts with L194 and Y195 of its binding partner Bcl-X_L_. Previous point mutation studies of these two Bad peptide residues (A5G and F23A) demonstrated ∼4 fold less binding affinity compared with wild type peptide also suggests the important role of these residues for binding [36]. Previous computational study also recognized these two residues as probable hot spots of BH3 peptides for Bcl-2 family proteins binding [44]. These strong interactions of A5 and F23 could be the possible reasons for the extended alpha helical nature of the Bad peptide appeared in the Bcl-XL/Bad peptide simulation (Figure S1B).

Unlike MDM2 binding pocket which is mostly constructed by hydrophobic residues, Bcl-X_L_ surface is composed of both hydrophobic and charged residues. The interior pocket is hydrophobic and the wall of the binding pocket is composed of charged residues. The Bad and less extent Bak peptides are having several charged residues to complement the charge environment of its binding partner. R10 of Bad peptide and R5 of Bak peptide forms stable hydrogen bonds with E129 residue of Bcl-X_L_. Salt bridge analysis incorporated in *vmd program suite* [64] was utilized to determine salt bridge forming pairs between protein and peptide complexes. This analysis revealed that salt bridges can be formed between R10 (Bad)/R5 (Bak) and E129 of Bcl-X_L_. Another arginine residue (R13) of Bad peptide form hydrophilic interactions with D133 and also forms salt bridge. The equivalent residue in Bak peptide is an alanine (A8) also contributes favorably (−2 kcal/mol) by forming close van der Waals contacts with L130 and R139 residues of Bcl-X_L_.

Several structural studies by experimental, computational methodologies and peptide mimitics as well as inhibitors of MDM2/p53 binding demonstrated that p53 contains three important hydrophobic residues (F19, W23 and L26). Our residual decomposition of MDM2/p53 results also depicted the same (Table 4). The residual contribution analysis of Bcl-X_L_/SN15 simulation postulated that indeed these three residues of the SN15 peptide are major contributors for binding with protein (Table 7). These three hydrophobic residues of SN15 occupy three hydrophobic pockets (p2–p4) in a similar manner as BH3 peptides (Figure 1). The residue L26 of SN15 superimposes well on equivalent residue leucine which is well conserved in all the BH3 peptides. It occupies the p2 binding pocket and forms van der Waals interactions in a similar way as BH3 peptides and contributes in same level for binding with Bcl-X_L_ (∼−5 kcal/mol). The central hydrophobic residue tryptophan (W23) of SN15 resides at the p3 hydrophobic binding pocket and interacts strongly with surrounding hydrophobic residues like F97 and A142. A stable hydrogen bond is observed between epsilon nitrogen atom of tryptophan and main chain carbonyl of E96. This could be the possible reason for larger contribution of binding energy of this residue for complex formation. The point mutation (W23A) simulation results produced around 10 kcal/mol less enthalpy (ΔH) compare to the wild type peptide simulation which is in good agreement with experimental data (Table 2 and Table S3). Our results postulated that tryptophan is the optimized option for binding at p3 hydrophobic pocket than methionine (Bad) or isoleucine (Bak). The Bcl-X_L_ p4 hydrophobic pocket formed by F97 and V141 residues provides space for another hydrophobic residue (F19) of SN15. Though the phenyalanine is present in both SN15 and Bad peptides the relative contribution of binding from SN15 is less (∼2 kcal/mol). In the case of SN15 the side chain of F19 slightly dislocates from the p4 pocket due to the relative binding orientation of the peptide. This could be the probable reason for the lower contribution of SN15 at particular interaction.

**Table 7:** Residual contribution of BH3 (Bad, Bak) and SN15 peptides for complex formation with Bcl-X_L_.

Bad	Energy contribution (kcal/mol	Bak	Energy contribution (kcal/mol	SN15^a^	Energy contribution (kcal/mol
N1	1.65				
L2	−2.29				
W3	−0.14				
A4	−0.63				
A5	−2.81				
Q6	−2.28	G1	3.44		
R7	−0.68	Q2	−0.96		
**Y8**	**−2.88**	**V3**	**−4.22**		
G9	−1.67	G4	−1.04	N29	2.66
R10	−4.73	R5	−3.4	E28	1.16
E11	0.97	Q6	−2.82	P27	−1.98
**L12**	**−3.3**	**L7**	**−4.79**	**L26**	**−4.95**
R13	−6.1	A8	−2	L25	−1.68
R14	−0.69	I9	−0.52	K24	−0.46
**M15**	**−3.22**	**I10**	**−4.27**	**W23**	**−6.88**
S16	1.98	G11	−0.79	L22	−2.1
D17	0.74	D12	−0.56	D21	1.39
E18	1.2	D13	0.92	S20	−2.56
**F19**	**−5.05**	**I14**	**−1.7**	**F19**	**−2.64**
V20	−1.54	N15	−0.72	T18	−0.19
D21	0.82	R16	−0.6	E17	0.53
S22	1.35			Q16	−5.72
F23	−1.99			S15	2.17
K24	−0.39				
K25	0.17				

a SN15 residues shown in reverse direction based on alignment with BH3 peptides (refer Figure 1E).

In addition to these three crucial hot spot residues, several other hydrophobic residues present on the SN15 peptide forms van der Waals interactions with Bcl-X_L_ binding pocket. Residue L22 which precedes important hydrophobic residue W23 shows favorable contribution to binding by forming close contacts with R139. Another hydrophobic residue L25 forms van der Waals contacts with L130 and R139 residues of Bcl-X_L_. L25 is present in an equivalent position as A8 of Bak peptide which exhibited similar type of interactions with the protein. P27 of SN15 peptide interacts considerably strong with the Bcl-X_L_ binding pocket by forming both hydrophobic and hydrogen bond interactions. The proline ring resides on the top of the p1 pocket and form hydrophobic interactions with side chains of L112, V126 and F146. The main chain P27 carbonyl forms a stable hydrogen bond interaction with Y101 side chain hydroxyl which is present on BH3 binding region of Bcl-X_L_.

Besides hydrophobic interaction ability of SN15 with Bcl-X_L_, several hydrophilic interactions were observed (Figure S3). The residue S20 contributed −2.5 kcal/mol for binding, constituting hydrogen bond with R100 of the protein. This hydrogen bond is observed between side chains of the both residues. Another residue Q16 forms several hydrogen bond interactions with E96, R100 of α2 helix. Though the SN15 peptide forms several hydrophilic residues, it lacks the charged residues at appropriate positions like Bad/Bak. SN15 peptide has a negatively charged residue (D21) that is conserved even in Bad and Bak peptides. Although, it is in a conserved position, due to the binding orientation of SN15 peptide the distance between the complimentary residue (R139) of Bcl-X_L_ and D21 of SN15 is high and is unable to reach and establish either hydrogen bond or salt bridge. Salt bridge as well as hydrogen bonding interactions was observed with aspartate residue (D12) which is present at equivalent position of Bak peptide. The calculated minimum distance between the side chains of aspartate residue of SN15, Bak (D21, D12 respectively) and arginine (R139) residue of Bcl-X_L_ is around 0.7 nm which is not optimal for any type of interaction. In the case of Bak, though the side chains are far at initial stages of the simulation but around 2 ns time the distance of the side chains reduced to around 0.2 nm and is sustained throughout the simulation (Figure S4). This reveals that SN15 peptide does not possess any charge complementary residues which can interact strongly with the charged Bcl-X_L_ binding pocket. This could be the possible reason for negative electrostatic values (ΔELE) obtained for the Bcl-X_L_/SN15 peptide simulation.

### Hot spot residues of Bcl-X_L_ involved in binding with BH3/SN15 peptides

Major contributors which contribute ≥−1 kcal/mol for complex formation were considered as hot spots of Bcl-X_L_ for the heterodimer formation (Figure S5A–5C). Although several residues are important for all the complexes, few differences also were noticed. These differences largely arise due to the length and helical nature as well as binding orientation of the different peptides (Figure 4B–4D). The Bad peptide covers maximum surface of the Bcl-X_L_ binding pocket and is composed of both hydrophobic as well as charged residues. Bak and SN15 peptides occupy comparatively less space due to their length. Nevertheless, all three peptides (Bad, Bak and SN15) interact with the conserved hydrophobic pocket residues. The p1 hydrophobic pocket residues F105, V126 and F146 are involved in interactions with h1 hydrophobic residues of the BH3 peptides and P27 of the SN15 peptide. All three residues are consistently engaged in favorable binding energy with all the peptides. Though SN15 lacks hydrophobic residue at equivalent position of h1 on BH3 peptides, P27 of SN15 shields the p1 site and forms hydrophobic interactions with these three residues. In the case of F146, it contributes little less than the benchmark contribution with SN15 peptide (−0.8 kcal/mol). Another notable difference is Y8 residue of Bad peptide establishes interaction with L112 while Bak and SN15 interacts with L108 (Table 7). The p2 hydrophobic pocket residues, F97 and L130 are consistently involved in favorable binding with all three peptide hydrophobic conserved leucine residue. Other two hydrophobic residues also contributed considerably with all the peptides.

**Figure 5:**
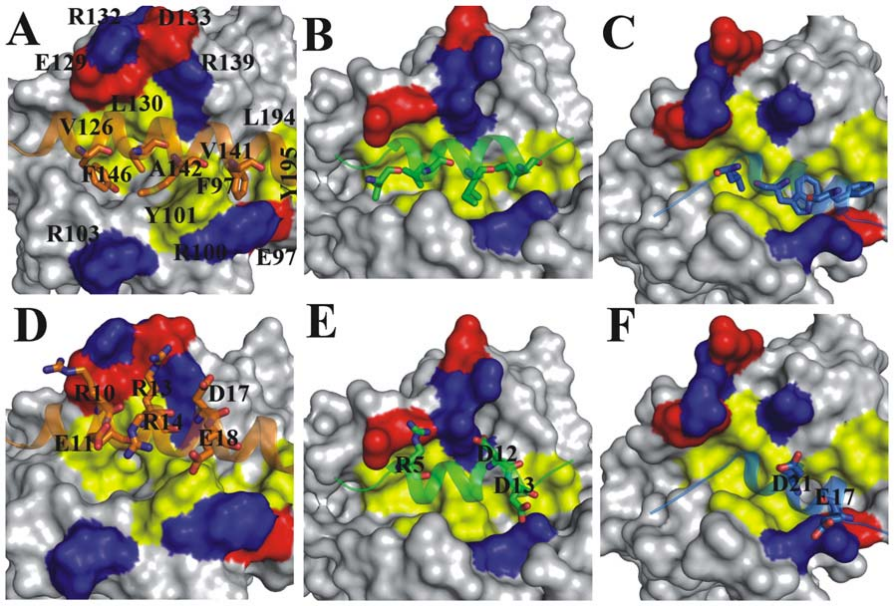
Hydrophobic and charged surface of Bcl-X_L_. The shallow hydrophobic pocket of Bcl-X_L_ is shown with yellow color, positive and negatively charged residues present on the walls of the pocket represented with blue and red, respectively. The hydrophobic residues of peptides h1–h4 of Bad (A), Bak (B) and h2–h4 of SN15 (C) are highlighted as sticks which lock with hydrophobic pockets of Bcl-X_L_. Complimentary charged residues on Bad (D), Bak (E), SN15 (F) are highlighted as sticks and labeled accordingly.

Both Bad and Bak peptides consists of two negatively charged successive amino acids (D17, E18 in Bad and D12 and D13 in Bak). These residues extend their side chains and cover both sides of the Bcl-X_L_ binding pocket charged walls (Figure 5D and 5E). They superimpose well in both the peptide binding conformations and are suspected to form hydrogen bond as well as salt bridge interactions with R139 and R100, respectively. But only in the case of Bak peptide both aspartate residues interact with arginine residues by either hydrogen bond interactions or salt bridges. This observation was clearly reflected in the residual decomposition analysis (Table 7). Both arginines R100 and R139 contributed predominantly (−3.5 and −5.1 kcal/mol, respectively), but the Bad peptide is not able to interact with these residues. The possible reason could be due to unfavorable interactions with R139 because of close proximity to another positively charged residue R13 on Bad peptide which causes charge repulsion. E18 is unable to form any type of interactions with R100; instead it forms a hydrogen bonding interaction with Y101. Residue Q16 of SN15 forms 2–3 hydrogen bond interactions with Bcl-X_L_. Main chain carbonyl group interacts with R100 and its side chain form hydrogen bonds with E96 side chain of the protein. Because of these two interactions E96 and R100 contributed favorably for complex formation (Figure S3).

### Common features of p53 (SN15) responsible for dual target binding

Residual decomposition and hot spot recognition for the complex formation revealed that several hydrophobic residues of the BH3 and SN15 peptides are forming similar type of interactions with Bcl-X_L_ (Figure 5). The three hydrophobic pillars of p53 (F19, W23 and L26) which are crucial for MDM2/p53 binding recognition are indeed important in complex formation with Bcl-X_L_. All these three residues occupy 3 out of 4 conserved hydrophobic pockets (p2–p4) of the Bcl-2 family proteins. Previously, attempts were made to develop potent Bcl-X_L_ inhibitors based α-helical peptidomimetics taking into consideration of three hydrophobic residues such as L7, I10 and I14 (i, i+3, and i+7) of Bak peptide [65], [66]. These mimetics developed from the BH3 peptide demonstrated binding towards Bcl-X_L_ and also with MDM2. Our recent finding revealed that even MDM2 potent inhibitor nutlin-3 and PMI (p53 mutant peptide) bind with Bcl-2 family proteins [67]. These observations suggest that though globular folding pattern of MDM2 and Bcl-2 proteins is different they share similar binding with p53.

**Figure 6:**
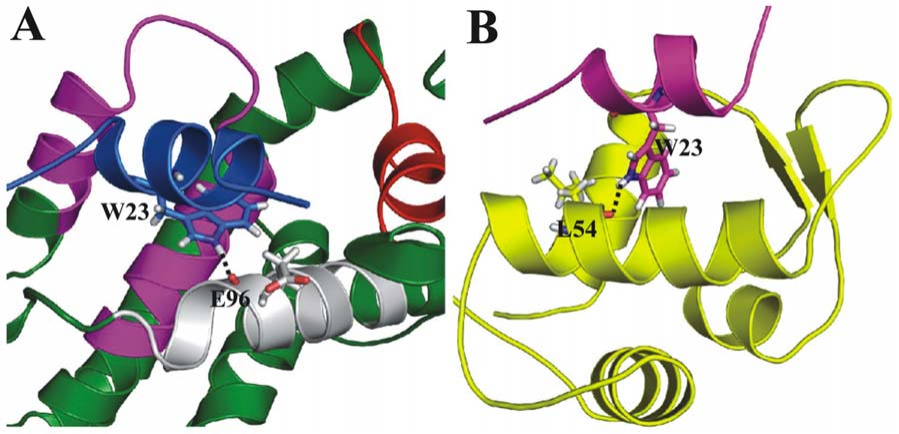
Hydrogen bonding pattern of p53 tryptophan residue. W23 residue of p53 or SN15 forms stable hydrogen bonding interaction with main chain carbonyl of E96 of Bcl-X_L_ are shown (A). Hydrogen bond interaction between W23 of p53 and main chain carbonyl of L54 residue of MDM2 are shown (B). Hydrogen bond represented with black dotted line.

Another important similarity in binding pattern of p53 with Bcl-X_L_ and MDM2 (Figure 6) is the stable hydrogen bond interaction between epsilon nitrogen atom of tryptophan (W23) side chain and main chain carbonyl of E96 (Bcl-X_L_), L54 (MDM2). Our simulations results in the present study postulated that P27 of SN15 covers the first hydrophobic pocket (p1) and forms van der Waals interactions with L112, V126 and F146. Recent crystal structures of MDM2 and MDMX with PMI (p53 based mutant peptide inhibitors) revealed that both the protein surfaces have an extra 4th hydrophobic pocket. The proline residue which is present at C-terminal end of PMI (TSFAEYWNLLSP) occupies this extra binding pocket (Figure S6) of MDMX formed by V49, M53, Y99 and L102. In MDM2 the equivalent residues are M50, L54, Y100 and I103. Surprisingly this pocket is unable to accommodate the proline residue of PMI due to the Y100 conformation. The point mutation studies clearly indicated that the proline change affects negatively on binding with MDMX protein. Despite these differences it's clear that these two proteins have 4th hydrophobic pocket similar as Bcl-2 proteins.

In conclusion, our present MD simulations coupled with binding free energy calculations corroborated with our previous NMR studies of binding pattern of SN15 with Bcl-X_L_. Residual decomposition results demonstrated similarities in the binding pattern between the BH3 and SN15 with Bcl-X_L_ protein. Key hydrophobic residues of p53 peptide (F19, W23 and L26), which are crucial for MDM2 binding occupy the hydrophobic patch of Bcl-X_L_ in an identical manner as Bad and Bak peptides. Though the SN15 satisfies the hydrophobic contribution similar to BH3 peptides, lack of charged interactions with the either sides of Bcl-X_L_ binding cavity walls leads to a modest binding affinity. The tryptophan residue of SN15 contributes dominantly for complex formation and forms both hydrophobic and hydrogen bonding interactions at third hydrophobic pocket of Bcl-X_L_. This residue contributes better than equivalent residues of BH3 peptides (M15, I10 of Bad and Bak peptides respectively). Analogous interaction pattern was observed between W23 of p53 and MDM2. This anchoring interaction of tryptophan in both complexes could be one of the reasons for susceptibility for mutational changes which causes decrease or total loss of binding [50], [59]. Recent evidence of nutlin-3 binding in Bcl-X_L_ substantiated the importance of tryptophan or tryptophan mimics for dual target binding [67]. Our results provide insights into the molecular basis for recognition of p53 peptide in MDM2 and Bcl-X_L_ and could be helpful to develop inhibitors which can bind to both targets.

## Supporting Information

Figure S1 **Secondary structural characteristics calculated using DSSP method in time dependent manner for MDM2/p53 (A), Bcl-XL/Bad (B), Bcl-XL/Bak (C), and Bcl-XL/SN15W23A (D) complexes.** Initial and final conformations of protein represented in cartoon style and secondary structures are labeled. The helix length of the peptides are represented with starting and ending residues of helix and highlighted by arrows. Secondary structural features also labeled accordingly to show the stability of the simulations.
(PDF)

Figure S2 **Intra-molecular hydrogen bonds calculated for the p53 (A), SN15 (B), Bad (C), and Bak (D) to understand the stability of the helical content of the peptides.** All the four peptides demonstrated stable and in several cases increased intra-molecular hydrogen bonding interactions.
(PDF)

Figure S3 **Inter-molecular hydrogen bond interactions observed between Bcl-XL and SN15 peptide.** Interacting residues are highlighted with sticks and hydrogen bonds represented with dashed line.
(PDF)

Figure S4 **The minimum distance measured between side chains of D21 (SN15), D12 (Bak) and R139 of Bcl-XL.**
(PDF)

Figure S5 **Residual decomposition and energy contribution of each residue in complex simulations of Bcl-XL/Bad (A), Bcl-XL/Bak (B), Bcl-XL/SN15 (C), and MDM2/p53 (D).** The straight line in graph represents the missing residues (45–84) which presents on long loop between α1 and α2 helices of Bcl-XL protein.
(PDF)

Figure S6 **The p53 peptidomimetic (PMI) interaction pattern with MDM2 (A), and MDMX (B).** The trio hydrophobic residues and terminal proline residues of PMI are shown as sticks and labeled. The hypothetical fourth hydrophobic pocket forming residues in MDM2 and MDMX are also highlighted as sticks.
(PDF)

Table S1 **Components of binding free energy (in kcal/mol) of Bcl-XL with Bad peptide.**
(PDF)

Table S2 **Components of binding free energy (in kcal/mol) of Bcl-XL with Bak peptide.**
(PDF)

Table S3 **Components of binding free energy (in kcal/mol) of Bcl-XL with SN15W23A peptide.**
(PDF)
