# Supplementary figures and images for: Molecular Basis of Bcl-XL-p53 Interaction: Insights from Molecular Dynamics Simulations
Source: PLoS One. 2011 Oct 19;6(10):e26014. doi: 10.1371/journal.pone.0026014 (PMC3198449; doi:10.1371/journal.pone.0026014)

Figure S1A.

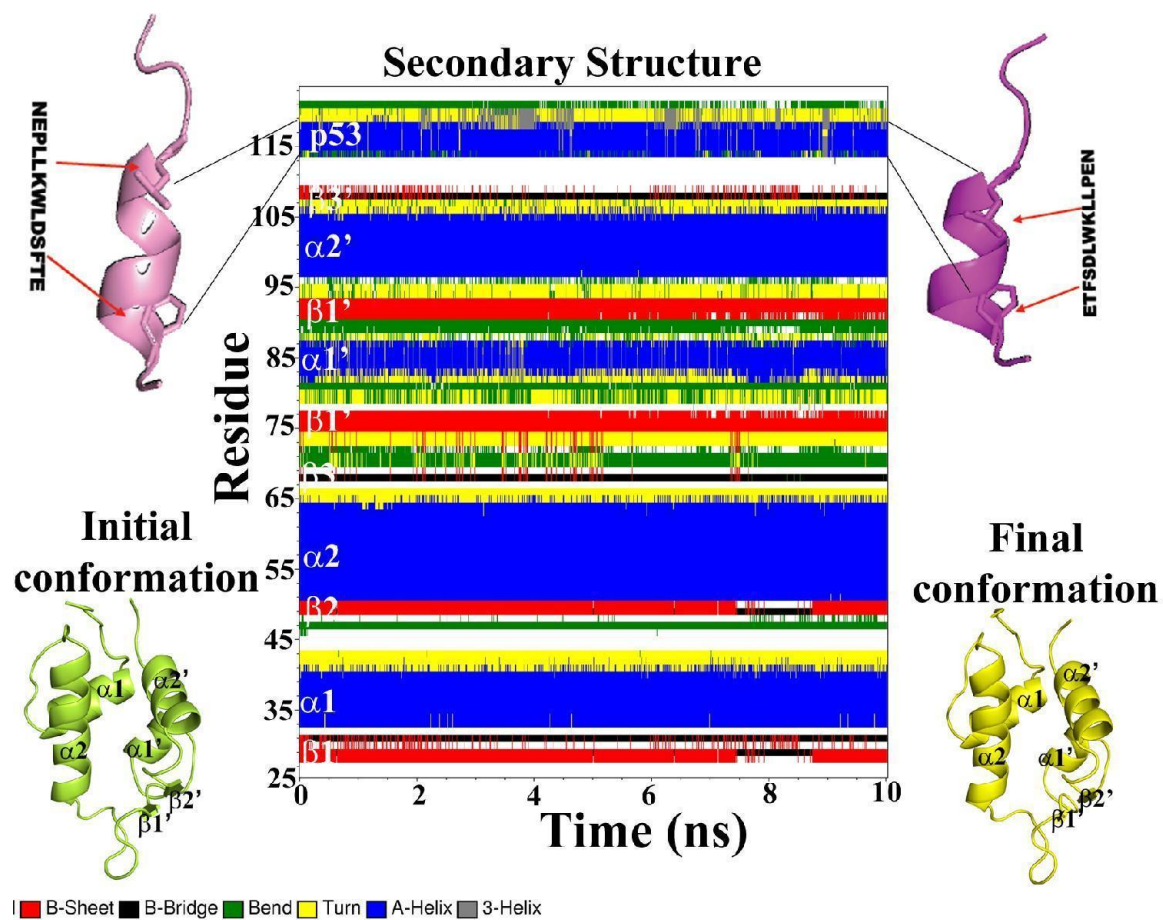

Figure S1B.

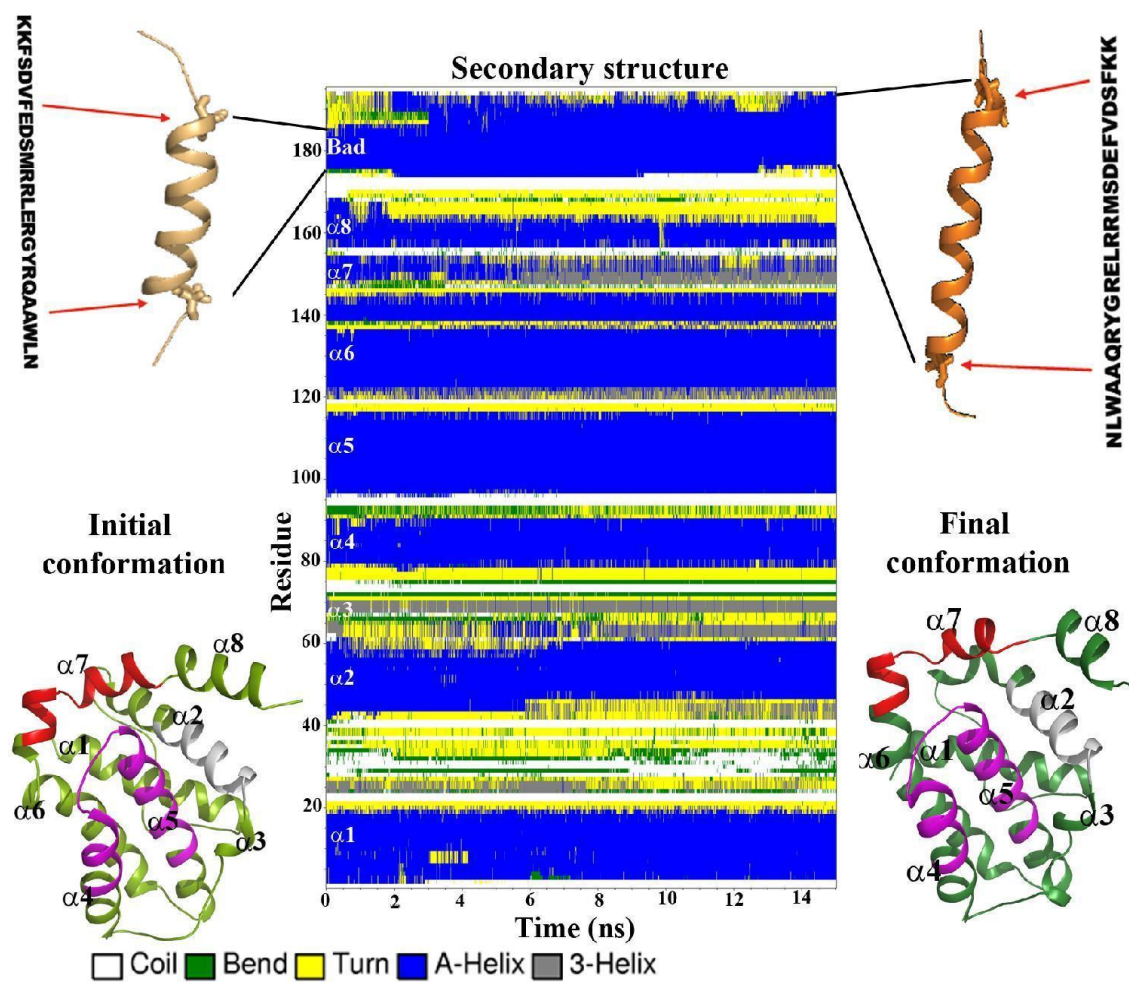

Figure S1C.

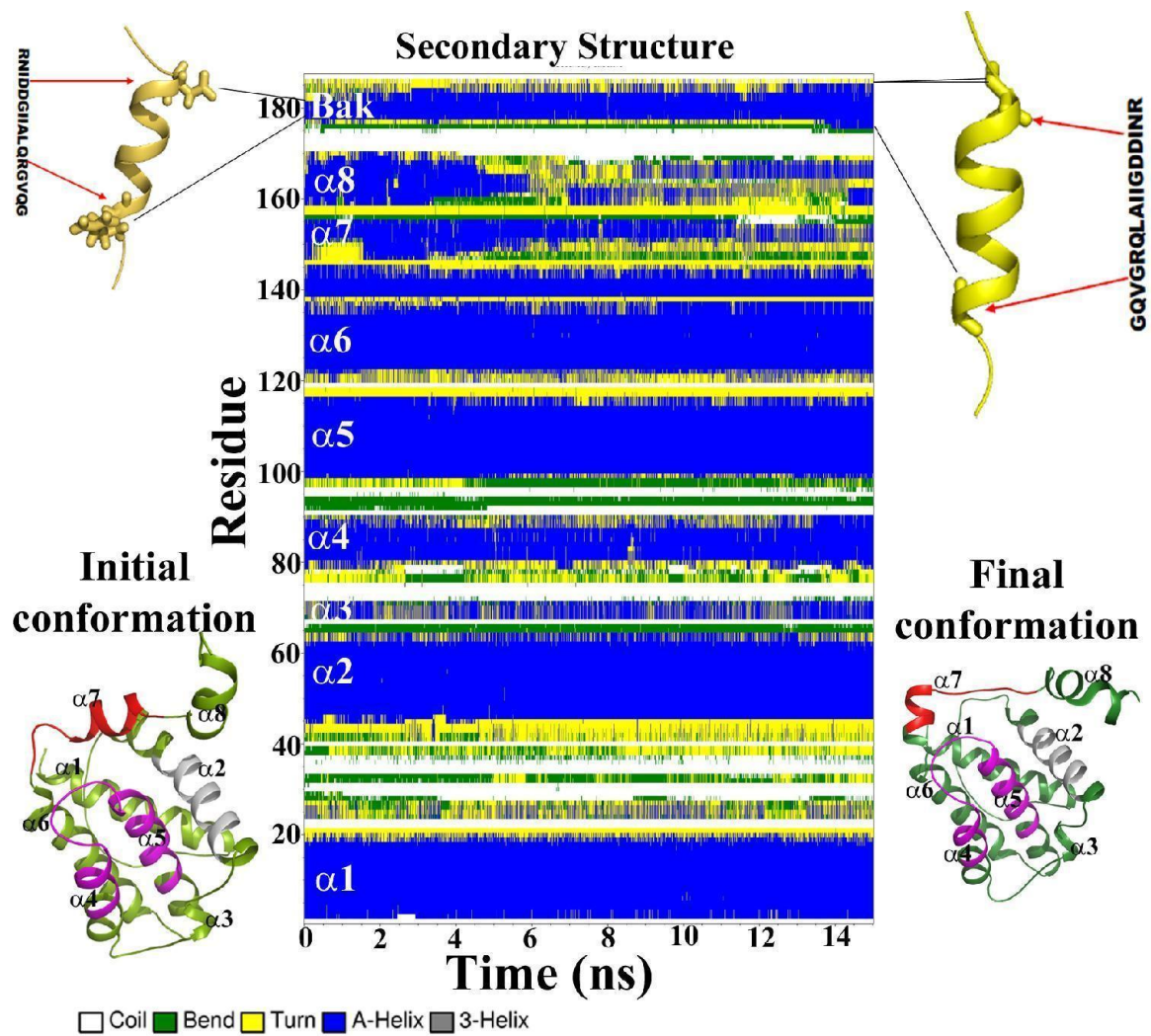

Figure S1D.

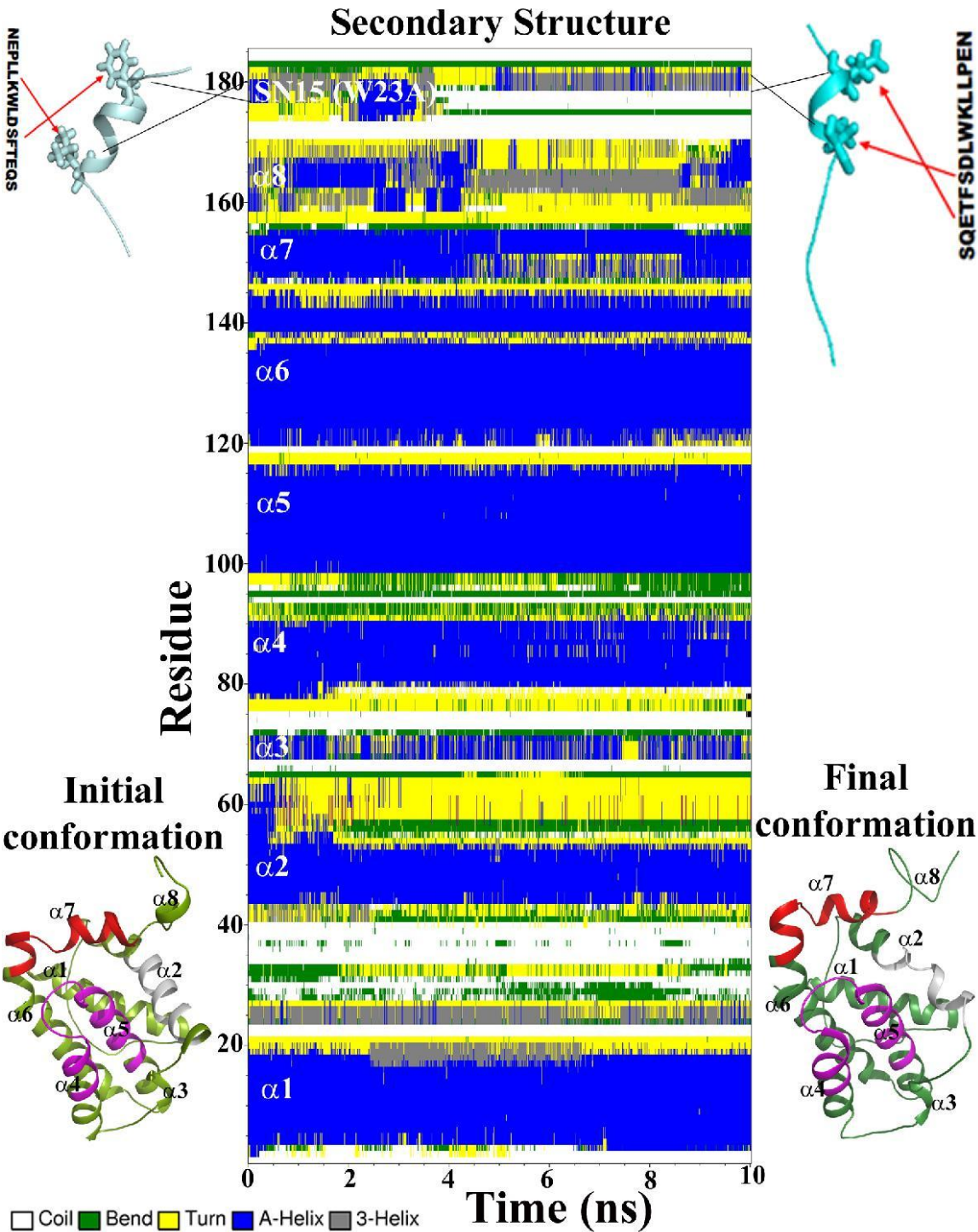

Supplement: Figure S1 — Secondary structural characteristics calculated using DSSP method in time dependent manner for MDM2/p53 (A), Bcl-XL/Bad (B), Bcl-XL/Bak (C), and Bcl-XL/SN15W23A (D) complexes. Initial and final conformations of protein represented in cartoon style and secondary structures are labeled. The helix length of the peptides are represented with starting and ending residues of helix and highlighted by arrows. Secondary structural features also labeled accordingly to show the stability of the simulations. (PDF) [file pone.0026014.s001.pdf]

Figure S2.

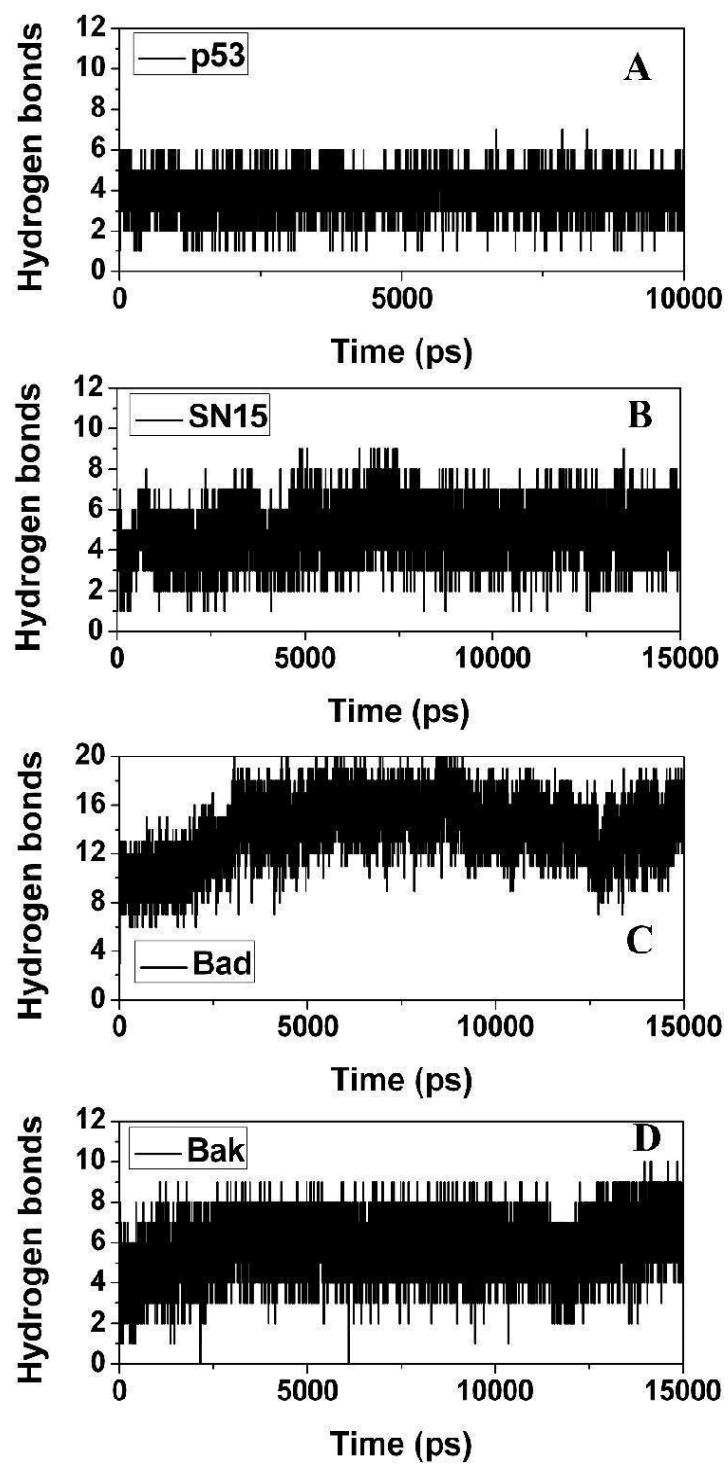

Supplement: Figure S2 — Intra-molecular hydrogen bonds calculated for the p53 (A), SN15 (B), Bad (C), and Bak (D) to understand the stability of the helical content of the peptides. All the four peptides demonstrated stable and in several cases increased intra-molecular hydrogen bonding interactions. (PDF) [file pone.0026014.s002.pdf]

Figure S3.

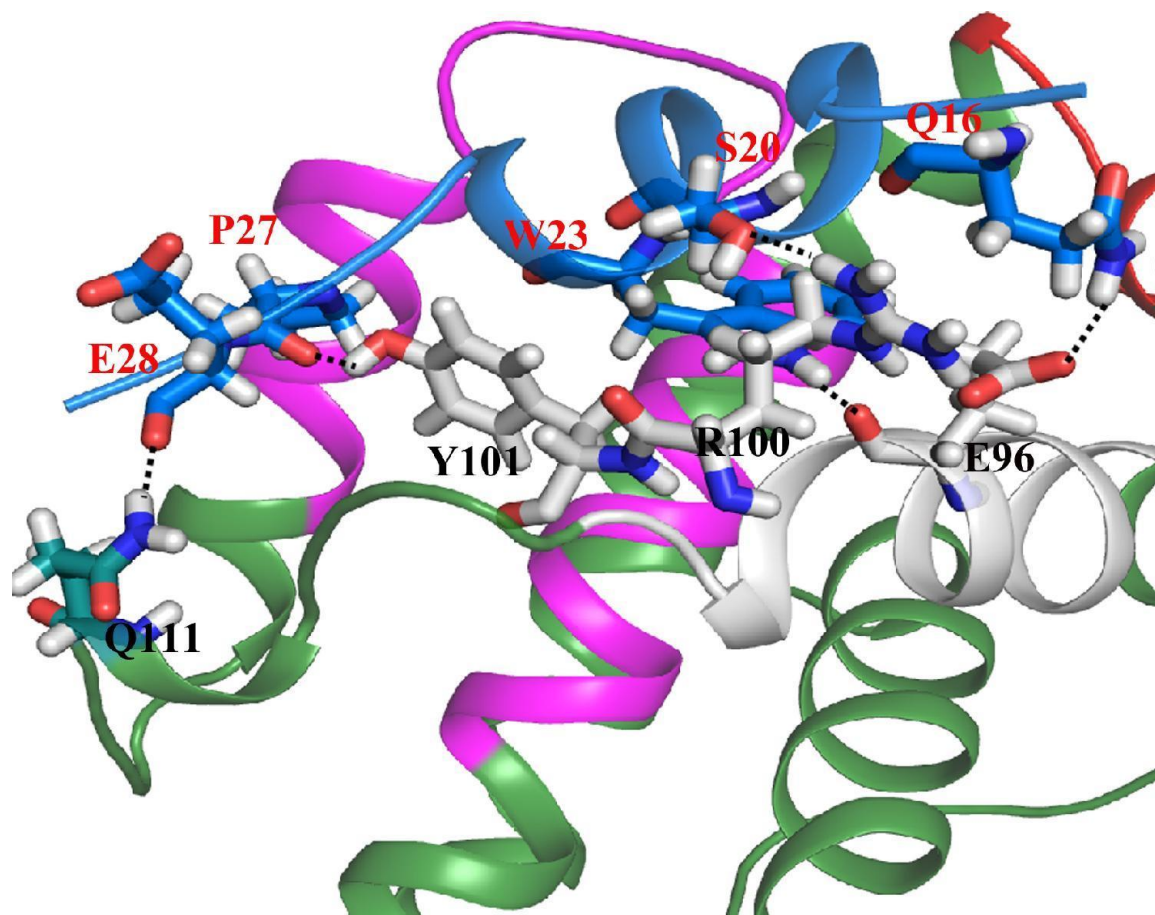

Supplement: Figure S3 — Inter-molecular hydrogen bond interactions observed between Bcl-XL and SN15 peptide. Interacting residues are highlighted with sticks and hydrogen bonds represented with dashed line. (PDF) [file pone.0026014.s003.pdf]

Figure S4.

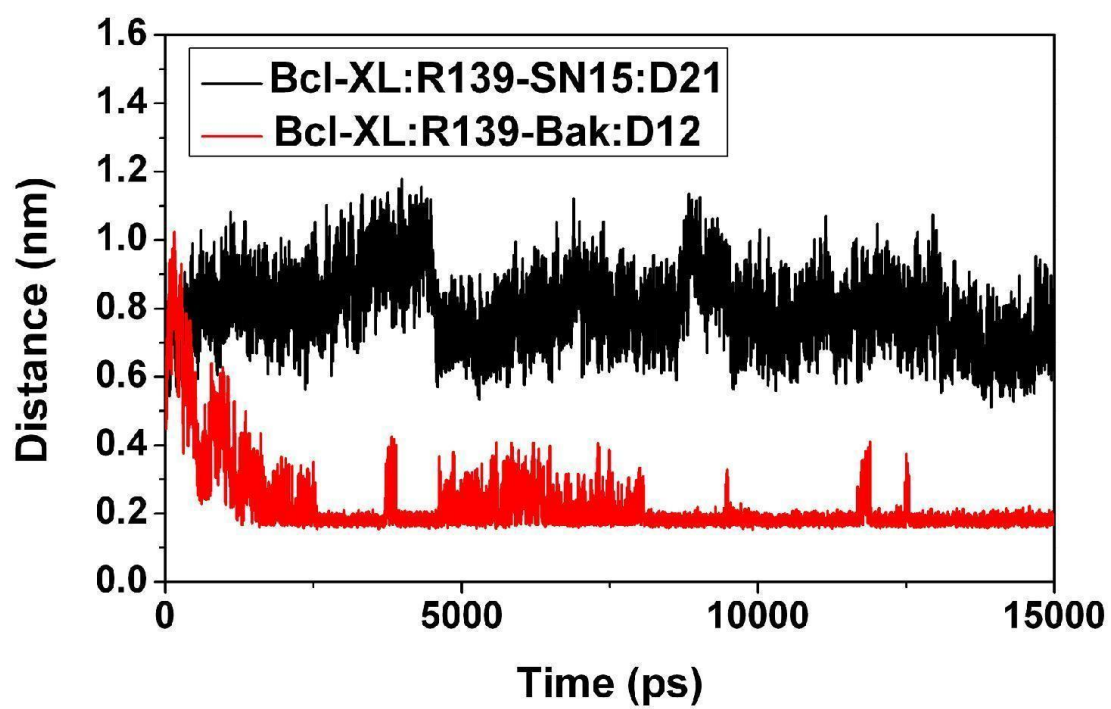

Supplement: Figure S4 — The minimum distance measured between side chains of D21 (SN15), D12 (Bak) and R139 of Bcl-XL. (PDF) [file pone.0026014.s004.pdf]

Figure S5A.

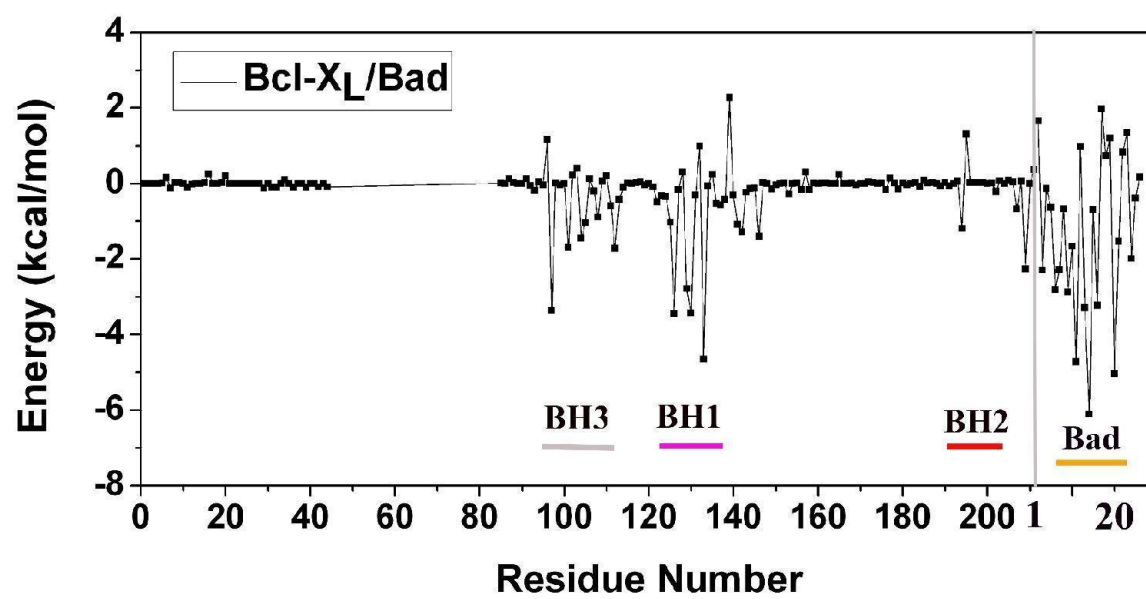

Figure S5B.

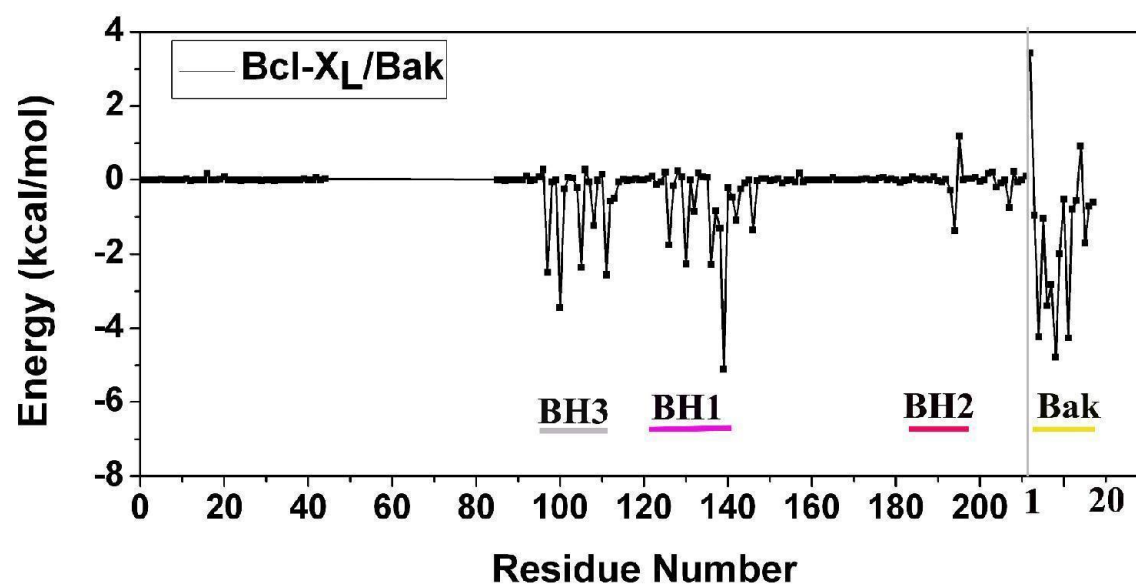

Figure S5C.

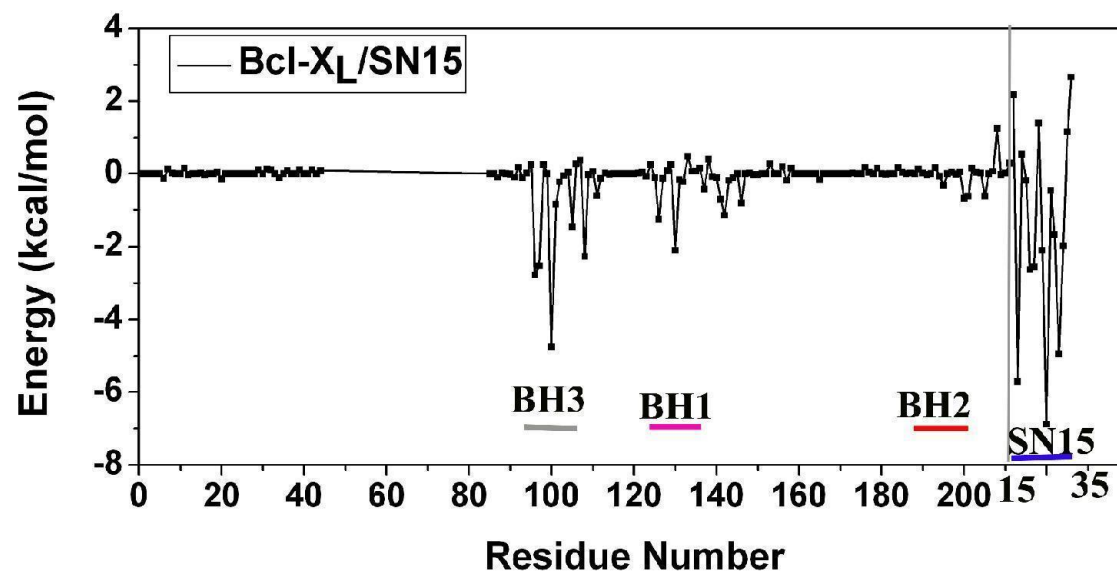

Figure S5D.

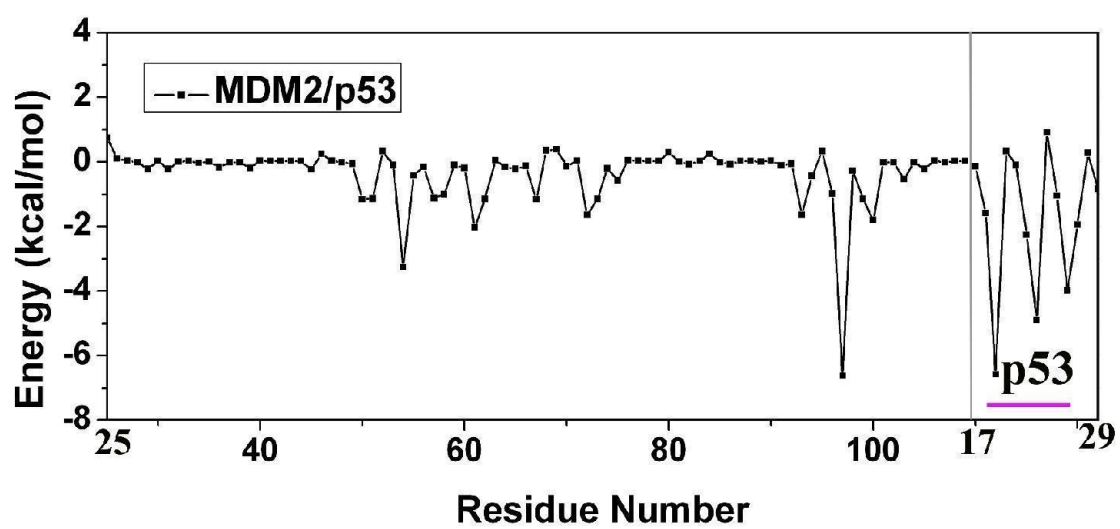

Supplement: Figure S5 — Residual decomposition and energy contribution of each residue in complex simulations of Bcl-XL/Bad (A), Bcl-XL/Bak (B), Bcl-XL/SN15 (C), and MDM2/p53 (D). The straight line in graph represents the missing residues (45–84) which presents on long loop between α1 and α2 helices of Bcl-XL protein. (PDF) [file pone.0026014.s005.pdf]

Figure S6.

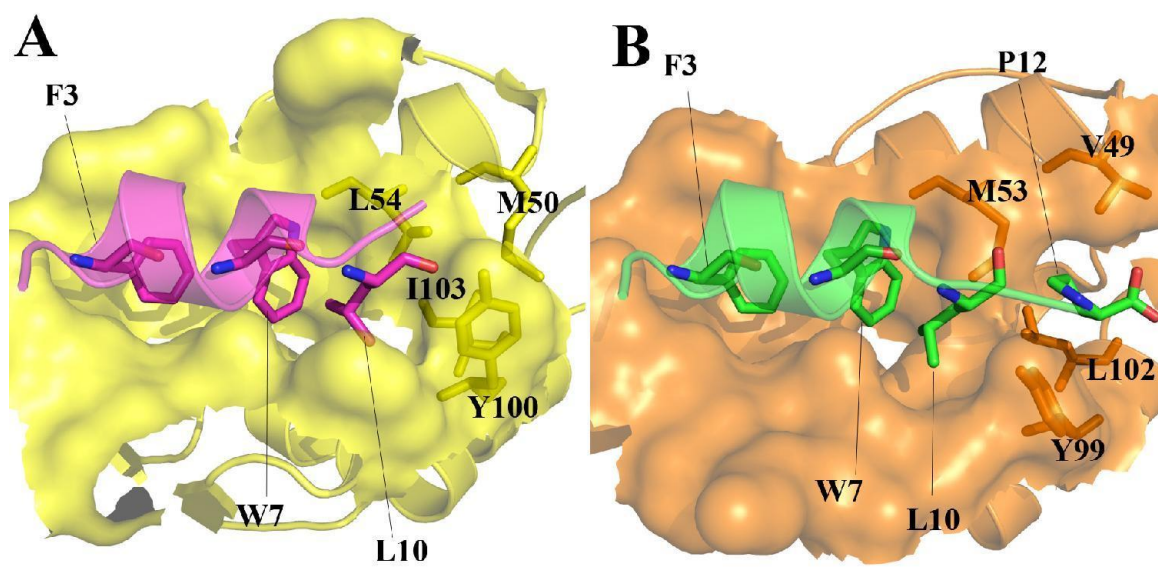

Supplement: Figure S6 — The p53 peptidomimetic (PMI) interaction pattern with MDM2 (A), and MDMX (B). The trio hydrophobic residues and terminal proline residues of PMI are shown as sticks and labeled. The hypothetical fourth hydrophobic pocket forming residues in MDM2 and MDMX are also highlighted as sticks. (PDF) [file pone.0026014.s006.pdf]
