# Supplementary material for: Molecular Basis of Bcl-XL-p53 Interaction: Insights from Molecular Dynamics Simulations
Source: PLoS One. 2011 Oct 19;6(10):e26014. doi: 10.1371/journal.pone.0026014 (PMC3198449; doi:10.1371/journal.pone.0026014)
Supplement: Table S1 — Components of binding free energy (in kcal/mol) of Bcl-XL with Bad peptide. (PDF) [file pone.0026014.s007.pdf]

Table S1. Components of binding free energy (in kcal/mol) of Bcl-XL with Bad peptide

	**Bcl-XL/Bad**		**Bcl-XL**		**Bad**		**Delta**
	**Average**	**Std**	**Average**	**Std**	**Average**	**Std**	
ELE	-6093.7	108.3	-4657.6	100.3	-938.9	42.0	-497.2
VDW	-749.5	25.7	-585.7	22.4	-46.2	8.0	-117.6
GAS	-3181.2	114.8	-2048.9	105.1	-517.5	43.4	-614.8
PBSUR	62.6	1.84	58.4	1.19	17.7	0.34	-13.4
PB	-3669.5	117.3	-3490.5	106.0	-736.3	39.2	557.3
PBSOL	-3606.9	115.9	-3432.1	105.1	-718.6	39.2	543.9
PBELE	-9763.2	38.6	-8148.1	32.9	-1675.2	9.54	60.0
**PBTOT**	-6788.1	50.6	-5481.0	46.9	-1236.2	16.5	**-71.0**
TSTRA	16.8	0	16.6	0	15.0	0	-14.9
TSROT	17.0	0	16.8	0	14.4	0	-14.2
TSVIB	2234.3	9.0	1934.0	7.0	324.5	2.2	-24.2
**TSTOT**	2268.1	9.0	1967.5	7.0	353.9	2.2	**-53.3**
$\Delta \mathbf{G_{bind}}$							**-17.7**

Electrostatic energy (ELE); van der Waals contribution (VDW); total gas phase energy (GAS); nonpolar contribution to the solvation free energy (PBSUR); the electrostatic contribution to the solvation free energy (PB); sum of nonpolar and polar contributions to solvation (PBSOL); sum of the electrostatic solvation free energy and MM electrostatic energy (PBELE); final estimated binding free energy (PBTOT); translational energy (TSTRA); rotational energy (TSROT); vibrational energy (TSVIB), total entropic contribution (TSTOT); binding free energy ($\Delta \mathbf{G_{bind}}$)
