# Supplementary material for: Molecular Basis of Bcl-XL-p53 Interaction: Insights from Molecular Dynamics Simulations
Source: PLoS One. 2011 Oct 19;6(10):e26014. doi: 10.1371/journal.pone.0026014 (PMC3198449; doi:10.1371/journal.pone.0026014)
Supplement: Table S2 — Components of binding free energy (in kcal/mol) of Bcl-XL with Bak peptide. (PDF) [file pone.0026014.s008.pdf]

Table S2. Components of binding free energy (in kcal/mol) of Bcl-XL with Bak peptide

	**Bcl-XL/Bak**		**Bcl-XL**		**Bak**		**Delta**
	**Average**	**Std**	**Average**	**Std**	**Average**	**Std**	
ELE	-5544.6	93.2	-4575.1	74.4	-639.7	24.1	-329.7
VDW	-687.3	24.0	-590.7	22.4	-6.1	7.0	-90.5
GAS	-2745.3	101.4	-1965.8	82.9	-359.3	26.7	-420.2
PBSUR	57.3	1.2	56.9	1.1	11.8	0.3	-11.4
PB	-3625.7	82.5	-3510.0	65.4	-485.4	23.6	369.6
PBSOL	-3568.4	81.9	-3453.0	64.8	-473.5	23.5	358.2
PBELE	-9170.3	35.4	-8085.1	33.5	-1125.1	6.1	39.9
**PBTOT**	-6313.8	50.6	-5418.8	47.5	-832.9	13.0	**-62.1**
TSTRA	16.7	0	16.6	0	14.5	0	-14.4
TSROT	16.9	0	16.8	0	13.5	0	-13.4
TSVIB	2105.9	6.1	1940.1	6.5	187.9	3.4	-22.0
**TSTOT**	2139.5	6.1	1973.5	6.5	215.8	3.5	**-49.8**
$\Delta \mathbf{G_{bind}}$							**-12.3**

Electrostatic energy (ELE); van der Waals contribution (VDW); total gas phase energy (GAS); nonpolar contribution to the solvation free energy (PBSUR); the electrostatic contribution to the solvation free energy (PB); sum of nonpolar and polar contributions to solvation (PBSOL); sum of the electrostatic solvation free energy and MM electrostatic energy (PBELE); final estimated binding free energy (PBTOT); translational energy (TSTRA); rotational energy (TSROT); vibrational energy (TSVIB), total entropic contribution (TSTOT); binding free energy ($\Delta \mathbf{G_{bind}}$)
