# Supplementary material for: Molecular Basis of Bcl-XL-p53 Interaction: Insights from Molecular Dynamics Simulations
Source: PLoS One. 2011 Oct 19;6(10):e26014. doi: 10.1371/journal.pone.0026014 (PMC3198449; doi:10.1371/journal.pone.0026014)
Supplement: Table S3 — Components of binding free energy (in kcal/mol) of Bcl-XL with SN15W23A peptide. (PDF) [file pone.0026014.s009.pdf]

Table S3. Components of binding free energy (in kcal/mol) of Bcl-XL with SN15W23A peptide

	**Bcl-XL-SN15W23A**		**Bcl-XL**		**SN15W23A**		**Delta**
	**Average**	**Std**	**Average**	**Std**	**Average**	**Std**	
ELE	-4878.4	77.2	-4762.8	78.7	-351.7	31.7	236.1
VDW	-647.8	26.1	-576.7	25.2	0.8	5.8	-71.9
GAS	-2041.7	87.2	-2129.9	87.6	-76.0	32.5	164.2
PBSUR	60.4	1.4	58.6	1.3	11.9	0.2	-10.0
PB	-3961.1	75.0	-3335.3	79.0	-436.8	30.2	-188.9
PBSOL	-3900.7	74.3	-3276.7	78.2	-425.0	30.1	-199.0
PBELE	-8839.5	29.9	-8098.1	28.6	-788.5	6.5	47.1
**PBTOT**	-5942.3	46.4	-5406.6	44.1	-501.0	12.5	**-34.8**
TSTRA	16.7	0	16.6	0	14.4	0	-14.3
TSROT	16.9	0	16.8	0	13.4	0	-13.3
TSVIB	2106.5	6.2	1939.0	5.2	176.8	2.8	-9.3
**TSTOT**	2140.1	6.2	1972.5	5.2	204.6	2.8	**-37.0**
ΔG_{bind}							**2.2**

Electrostatic energy (ELE); van der Waals contribution (VDW); total gas phase energy (GAS); nonpolar contribution to the solvation free energy (PBSUR); the electrostatic contribution to the solvation free energy (PB); sum of nonpolar and polar contributions to solvation (PBSOL); sum of the electrostatic solvation free energy and MM electrostatic energy (PBELE); final estimated binding free energy (PBTOT); translational energy (TSTRA); rotational energy (TSROT); vibrational energy (TSVIB), total entropic contribution (TSTOT); binding free energy (ΔG_{bind})
